# QRS-T-angle in Patients with ST-Segment Elevation Myocardial Infarction (STEMI) - a Comparison with Cardiac Magnetic Resonance Imaging

**DOI:** 10.7150/ijms.44312

**Published:** 2020-08-25

**Authors:** B. Zadeh, J. M. Wambach, M. Lambers, K. Nassenstein, C.J. Jensen, O. Bruder

**Affiliations:** 1 Department of Cardiology and Angiology, Contilia Heart and Vascular Center, Elisabeth-Krankenhaus Essen, Essen, Germany; 2 Ruhr University Bochum, Bochum, Germany; 3 Department of Diagnostic and Interventional Radiology and Neuroradiology, University Hospital Essen, Essen, Germany

**Keywords:** QRS-T angle, cardiovascular magnetic resonance, late gadolinium enhancement, ST-segment elevation myocardial infarction

## Abstract

**Background**: The QRS-T angle from the surface EKG is a promising prognostic marker in patients with coronary artery disease. Cardiovascular magnetic resonance (CMR) imaging with late gadolinium enhancement (LGE) offers high resolution imaging of myocardial damage. We investigated the association of the QRS-T angle and the extent of myocardial damage as assessed by LGE in patients with acute ST-segment myocardial infarction (STEMI)

**Methods**: 169 patients with STEMI obtained a standardized digital 12-lead EKG on admission for the calculation of the QRS-T angle and underwent CMR imaging for analysis of infarct size by LGE within the first week. Patients were divided into groups: (1) abnormal QRS-T angle ≥ 90 degree and (2) QRS-T angle < 90 degree.

**Results**: Patients with a QRS-T angle of 90 degree or more had larger infarcts (36.5±12.4 vs. 13.3±9.5; p<0.001) and lower ejection fraction (42.9±12.1% vs. 50.6±10.6%; p<0.001).

**Conclusion**: The extent of myocardial damage as measured by the gold standard LGE is associated with a larger QRS-T angle calculated from the surface EKG.

## Introduction

Early restoration of epicardial blood flow by percutaneous coronary intervention (PCI) or thrombolysis is the gold standard of ST-segment elevation myocardial infarction (STEMI) treatment 1, 2. Risk stratification for recurrent myocardial infarction, heart failure and sudden cardiac death is key to treatment in the chronic phase of survived acute STEMI 3, 4.

Apart from clinical risk scores, biomarkers and imaging it would be extremely helpful to establish a simple parameter from the routine surface ECG for risk stratification in acute STEMI 5.

The QRS-T angle represents the spatial angle between the QRS loop (depolarization) and T loop (repolarization) 6. A wide QRS-T angle (**≥**90°) seems to correlate with electrical instability and has been related to cardiac prognosis in several studies 7, 8.

Cardiovascular Magnetic Resonance (CMR) imaging following gadolinium contrast administration allows for high-resolution imaging of myocardial damage 9. Late gadolinium enhancement (LGE) distribution patterns differentiate between ischemic (infarct) and non-ischemic (inflammation, fibrosis) myocardial pathology 10. The extent of LGE is a strong and independent prognosticator in a variety of clinical settings 11, 12, 13. In myocardial infarction CMR detects small subendocardial infarcts, and the transmural extent of LGE is related to left ventricular remodeling and recovery of function after revascularization 14.

This study aims to investigate the correlation of the QRS-T-angle on the 12-lead surface ECG and the extent of myocardial damage in patients with acute STEMI as assessed by late gadolinium enhancement CMR.

## Methods

Consecutive patients admitted to the Department of Cardiology and Angiology at Elisabeth Hospital Essen from February 2015 until March 2017 with acute ST-segment elevation myocardial infarction (STEMI) were enrolled into the study. Patients obtained a standardized 12-lead digital ECG immediately on admission using a Schiller Cardiovit AT 102 plus®. Computerized values of QRS and T-wave axis were given by the Schiller AT 102 plus® software. The frontal QRS-T-angle was calculated as the absolute difference between the frontal QRS- and frontal T-wave axes. With a value greater than 180°, 360° were subtracted in order to give a continuous variable ranging from 0° to 180°.

CMR imaging was performed on a 1.5 Tesla MR System (Magnetom Avanto™, Siemens Medical Solutions, Erlangen, Germany) not later than 7 days after admission. MR sequences were acquired using a phased-array receiver coil during end-inspiratory breathholding and in line with the recommendations of the Society of Cardiovascular Magnetic Resonance (SCMR) 15. Contiguous short axis and three long axis cine images were acquired by using a steady-state free precession (SSFP) sequence. For Late Gadolinium Enhancement (LGE) imaging corresponding segmented inversion recovery gradient echo sequences (IR-GRE) short axis views were obtained every 10mm (slice thickness 6mm) covering the entire left ventricle. LGE imaging was performed at least 10 minutes following the i.v. administration of 0.15 mmol/kgBW gadoterate meglumine, adjusting the inversion time as described previously. In-plane resolution was typically 1.2 x 1.8 mm 16.

Cine and LGE images were evaluated blinded to the clinical data by two experienced observers in consensus. LV function was analyzed by outlining epicardial and endocardial borders on the short axis SSFP sequences. Papillary muscles were excluded from analysis. Left ventricular volumes and ejection fraction were derived from contour summation. The extent of late gadolinium enhancement was assessed on long and short axis contrast images by using the 17-segment model of the LV. Typical expansion of LGE in patients with acute myocardial infarction (STEMI) is defined as areas of high signal intensity progressing from the subendocardial myocardial layer. Areas with ischemic infarct scar were defined as enhanced with a signal intensity above at least five SD as compared to non-infarcted remote myocardium. The presence and transmurality of infarcts were scored using the 17-segment model and a 5-point scala per segment (0=0%, 1=1-25%, 2=26-50%, 3=51-75%, 4=76-100%). The total infarct size as expressed as percentage of LV mass was calculated as follows: sum of enhanced segments and their transmurality score (LGE score) divided by 17 segments multiplied by the maximum transmurality score 17.

Statistical analyses were performed with the Excel™ software (Version 14.6.7, Microsoft™). Depending on the distribution continuous variables were expressed as means with standard deviation. Patients were divided into two groups (QRST-angle of 90 degree or larger vs smaller than 90 degree). The two-sample t-test was chosen to analyze differences between these two groups. Pearson correlation and one-sample t-test were applied to investigate the correlation of QRS-T angle and CMR parameters in each group. P values <0.05 were considered as statistically significant.

## Results

From February 2015 to March 2017, 169 patients (125 male, 44 female, 60.8 ± 11.1 years) admitted for acute STEMI (121 anterior wall myocardial infarction, 43 inferior wall myocardial infarction) were enrolled.

Patients were divided into two groups: (1) patients with a frontal QRS-T angle of 90 degree or more (n=54), (2) patients with a frontal QRS-T angle of less than 90 degree (n=114) (Table 1).

Mean QRS-T angle in group 1 and group 2 was 125.8±38.3 and 33±21.1 respectively. Patients with a QRS-T angle of 90 or more were older (63.7±11.2 vs. 59.4±10.9; p<0.05) and the heart rate on admission EKG was higher (85±17 bpm vs. 77±17 bpm; p<0.05). There was no difference between the two groups with regards to maximum creatinkinase (group 1: 1452±1778.2 U/l vs. group 2: 1451±1741.1 U/l; p=0.99) and maximum troponine (group 1: 3.0±3.1 ng/ml vs. group 2: 3.4±4.0 ng/l; p=0.44).

**Figure 1:**
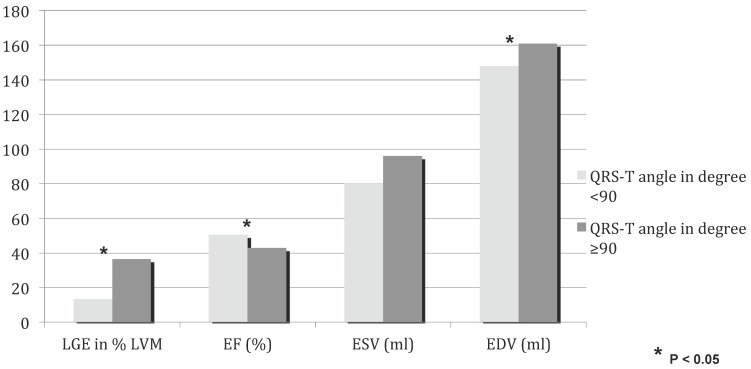
CMR parameters for QRS-T angle ≥90 (dark) and QRS-T angle <90 (bright). LGE late gadolinium enhancement, LVM left ventricular mass, EF ejection fraction, ESV end-systolic volume,EDV end-diastolic volume

**Figure 2:**
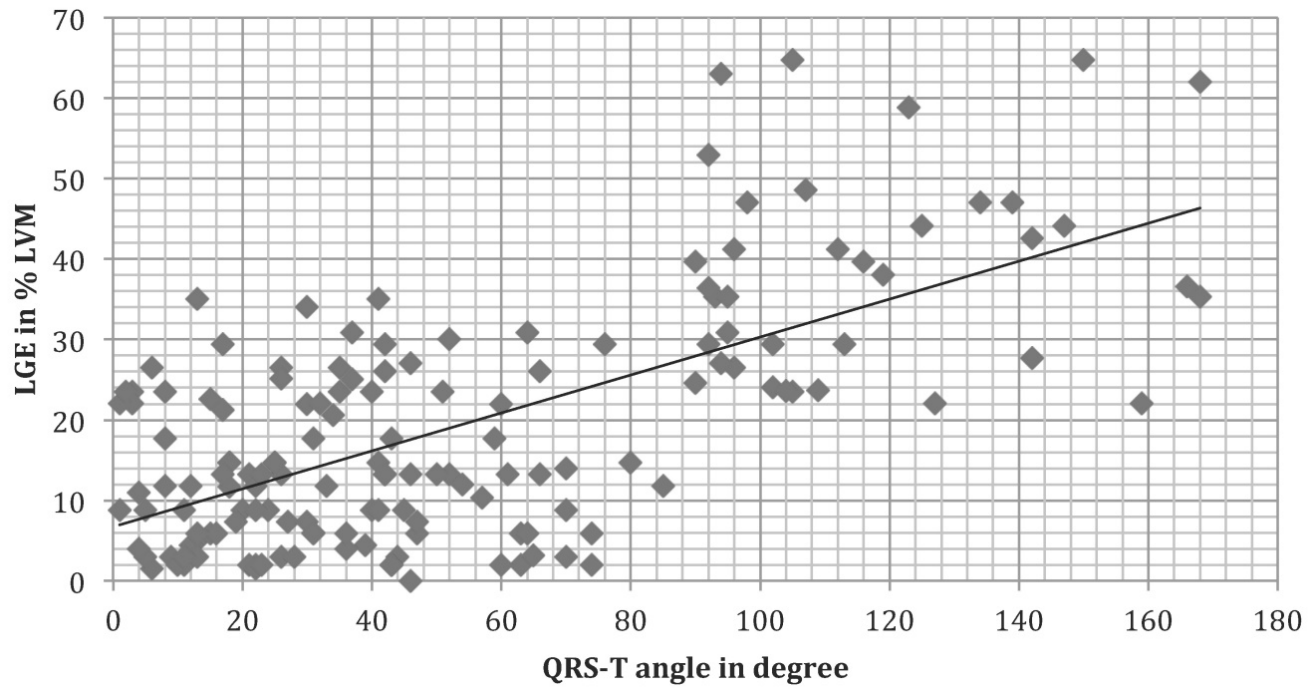
LGE score versus QRS-T angle. Pearson correlation coefficient was r=0.65936. LGE late gadolinium enhancement, LVM left ventricular mass

On CMR imaging (Figure 1, Table 1) ejection fraction was lower with a QRS-T angle of 90 degree or more (42.9±12.1% vs. 50.6±10.6%; p<0.001). 50 of 114 patients in group 2 had a normal left ventricular ejection fraction, whereas ejection fraction was 45% or less in 34 of 54 patients in group 1. The extent of myocardial necrosis as represented by percent late gadolinium enhancement of LV mass (LVM) was significantly (p<0.001) higher (36.5±12.4) in group 1 with a QRS-T angle of 90 or more as compared to group 2 (13.3±9.5). In addition, there is a moderate correlation of LGE percent LVM and the QRS-T angle (Figure 2, r=0.65936).

## Discussion

Our study shows that in a population of STEMI patients QRS-T angle on the surface EKG correlates with infarct size on contrast-enhanced CMR.

Previously several studies have been able to demonstrate the prognostic value of QRS-T angle in different patient populations. In a general Rotterdam population of 6,134 patients Kors et al. found an odds ratio of 5.6 for sudden cardiac death, which was superior to standard EKG indicators of adverse events such as ST depression and T wave inversion 18. Borleffs et al. detected that in a group of 412 ICD carriers a wide QRS-T angle was highly predictive of adequate ICD discharge and ventricular tachycardia 19. In 2705 consecutive patients with suspected NSTEMI QRS-T angle adds diagnostic accuracy as compared to standard EKG criteria of ischemia alone and predicts cardiac mortality 20. A frontal QRS-T angle of more than 90 degrees in 467 STEMI survivors with reduced ejection fraction demonstrated a strong and independent prognostic value 21.

Several ECG signs have been evaluated against distribution, transmurality and extent of late gadolinium enhancement on CMR following gadolinium contrast administration: In acute STEMI the resolution of ST-segment elevation, a marker of successful reperfusion therapy, relates to LGE and prognosis 22. A fragmentation of the QRS complex, however, does not predict late gadolinium enhancement in acute myocardial infarction 23. And ST-segment elevation in the right precordial leads indicates right ventricular involvement in patients with inferior wall myocardial infarction only in larger right ventricular infarcts if LGE of the right ventricle serves as reference standard 24. In chronic infarction, LGE identifies infarct scars in patients with clinically silent myocardial infarction that do no exhibit significant Q waves on the surface EKG 25, 26. Moon et al. found that in a group of 100 consecutive patients with chronic infarction the presence of Q waves was related to infarct size more than to infarct transmurality on late gadolinium enhancement CMR 27.

The concept of validating EKG parameters by scar (LGE) rather than left ventricular morphology, hypertrophy and function has also been investigated in the setting of non-ischemic cardiomyopathies: In hypertrophic cardiomyopathy late gadolinium enhancement is a strong and independent prognosticator of cardiac death 28, 29. Delcrè et al. reported a strong correlation of an EKG score including 9 independent markers and late gadolinium enhancement size in 257 patients with hypertrophic cardiomyopathy 30.

For a prognostic EKG marker that represents myocardial depolarization and repolarization in the way the QRS-T angle does, the correlation to myocardial pathology on CMR is particularly useful. To the best of our knowledge, this is the first study that relates QRS-T angle in patients with acute myocardial infarction (STEMI) to CMR function and scar.

Several non-invasive parameters that could help risk stratify patients with acute myocardial infarction are used in clinical routine. These include left ventricular ejection fraction, left ventricular volumes, several ECG parameters and detection of myocardial necrosis by contrast-enhanced cardiovascular magnetic resonance imaging. The ultimate goal in risk stratification is to identify patients who will suffer from sudden cardiac death and who need further protection, e.g. the implantation of cardioverter-defibrillator (ICD). However, up till now no single parameter has been successful to fulfill the task. In fact, due to the complex pathophysiology of ventricular arrhythmias a combination of non-invasive parameters might improve the prediction of ventricular arrhythmias in post acute MI patients. The QRS-T angle in patients with acute MI can be ubiquitously assessed and might add to clinical risk stratification in the future.

In conclusion, a QRS-T angle of 90 degrees or more is associated with lower ejection fraction and greater infarct size as assessed by contrast-enhanced CMR. Prospective studies in a larger STEMI patient population with clinical follow-up should be carried out in order to further investigate the prognostic impact of the QRS-T angle and its potential implication on clinical workflow.

## Figures and Tables

**Table 1:** Clinical and CMR Parameter for QRS-Tangle ≥90 vs. QRS-T angle <90

Parameters	QRS-T ≥ 90 (n = 54)	QRS-T < 90 (n = 114)	p - value
*Demographic Data*			
gender (w/m) in %	26/74	27/73	0.957
QRS-T angle (degree)	125.8 ± 38.3	33.0 ± 21.1	**<0.001**
age at infarction (years)	63.7 ± 1.2	59.4 ± 10.9	**0.019**
heart rate (bpm)	85.2 ± 17.3	76.6 ± 17.2	**0.003**
*CMR Data*			
ejection fraction, EF (%)	42.9 ± 12.1	50.6 ± 10.6	**<0.001**
ESV (ml)	96 ± 42	80 ± 32.1	0.089
EDV (ml)	161 ± 46	148 ± 39	**0.003**
pericardial effusion (mm)	3.1 ± 4.1	1.0 ± 2.1	**0.034**
LGE in % LV mass	36.5 ± 12.4	13.3 ± 9.5	**<0.001**
no reflow zone (number of patients)	11	14	0.121
*Clinical Data*			
ck maximum (U/l)	1452 ± 1778.2	1451 ± 1741.1	0.998
troponin maximum (ng/ml)	3 ± 3.1	3.4 ± 4.1	0.438
Cardiovascular risk factors			
arterial hypertension	43	65	**0.004**
family disposition	14	31	0.860
hyperlipidaemia	16	41	0.421
diabetes mellitus	9	19	0.996
obesity	20	35	0.414
smoking	31	64	0.888

LGE late gadolinium enhancement, LV left ventricular, ESV end-systolic volume, EDV end-diastolic volume
